# Effect of Ultrasound-Guided Percutaneous Neuromodulation of Sciatic Nerve on Hip Muscle Strength in Chronic Low Back Pain Sufferers: A Pilot Study

**DOI:** 10.3390/jcm11226672

**Published:** 2022-11-10

**Authors:** Roberto San-Emeterio-Iglesias, Blanca De-la-Cruz-Torres, Carlos Romero-Morales, Francisco Minaya-Muñoz

**Affiliations:** 1 Department of Physiotherapy, University Gimbernat-Cantabria, Aurelio García Cantalapiedra Street, s/n, 39300 Cantabria, Spain; 2 Department of Physiotherapy, University of Seville, Avicena Street, 41009 Seville, Spain; 3 Faculty of Sport Sciences, Universidad Europea de Madrid, Villaviciosa de Odón, 28670 Madrid, Spain; 4 MVClinic Institute, 28600 Madrid, Spain; 5 Faculty of Medicine, CEU San Pablo University, 28925 Madrid, Spain; 6 Physiotherapy Department, GAVA Medical Center, 28600 Madrid, Spain

**Keywords:** neuromodulation, hip, sciatic nerve, low back, muscle strength

## Abstract

**Background:** Limited hip internal rotation range of motion (IR-ROM) and hip abductor weakness are recognized in low back pain (LBP) sufferers. The main aim was to investigate the effect of a ultrasound (US)-guided percutaneous neuromodulation (PNM) technique on hip strength in people with LBP. A second purpose was to discover whether the location along the sciatic nerve, where percutaneous neuromodulation was applied, could influence the change of strength response in these patients. **Methods**: Sixty LBP sufferers were recruited and divided randomly into three groups. All participants received an isolated percutaneous electrical stimulation at one of three different locations of the sciatic nerve pathway (proximal, middle, and distal), depending on the assigned group. Pain intensity, hip passive IR-ROM, hip muscle strength, and the Oswestry disability index (ODI) were analyzed. All variables were calculated before the intervention and one week after the intervention. **Results**: All interventions significantly decreased pain intensity and improved the IR-ROMs, strength, and functionality after one week (*p* = 0.001). However, between-group (treatment x time) differences were reported for flexion strength in the non-intervention limb (*p* = 0.029) and ODI (*p* = 0.021), although the effect size was small (Eta^2^ = 0.1) in both cases. **Conclusions**: The application of an isolated intervention of the US-guided PNM technique may be a useful therapeutic tool to increase the hip muscle strength in patients with chronic LBP.

## 1. Introduction

Chronic low back pain (LBP) is one of the most common musculoskeletal pains [1] and has been the main origin of physical disability for the past three decades [2]. LBP is identified as a nonspecific, painful, or mechanical condition in the low back, buttocks, or hips [1]. Although there are many factors that may be the origin of a chronic LBP [3,4], a muscle hip disbalance has been recognized [5]. This disbalance is characterized by a limited hip internal rotation range of motion (IR-ROM) [6] and hip abductor weakness [7]. Hence, therapists have aimed attention on the relevance of hip muscle strength for the designing of rehabilitation protocols [4].

Considering that exercise programs have been considered as a main intervention for hip muscle strength [8], innovative therapeutic solutions may be incorporated to help achieve the same goal. Peripheral nerve stimulation (PNS) has recently become an important area in the field of rehabilitation, being a therapeutic method to pain management. Regarding chronic LBP, from the medical point of view, PNS has been applied through the implantation of peripheral nerve leads, with the target of stimulating either the peripheral nerve trunks or nerve roots within the lumbosacral plexus [9,10]. From the physiotherapeutic point of view, an area of growing interest is ultrasound (US)-guided Percutaneous Neuromodulation (PNM) [11]. With the possibility to apply this innovate neural therapy using an electrical current in the peripheral nerve territory, this therapy offers an extraordinary chance to treat chronic pain disorders. The US-guided PNM technique is based on the use of a low frequency current through an acupuncture needle that is situated next to the nerve or motor point of the muscle with ultrasound guidance [11]. According to the authors’ best knowledge, there is emerging scientific knowledge on the application of this procedure for a variety of clinical conditions, such as elbow pain [12], anterior knee pain [13], cubital tunnel syndrome [14], and lower back pain [15]. Prior research has reported positive results from the US-guided PNM technique on radial, cubital, femoral, and sciatic nerves [12,13,14,15]. Despite this, although most investigations have aimed attention on pain control, specific evidence also supports the application of this technique on the nerve, with the improvement of muscle performance, ROM, balance, or function [16,17,18,19,20]. Therefore, the authors hypothesize that neuromodulation (especially the US-guided PNM technique) may be used to obtain more therapeutic effects, including pain relief, in LBP sufferers.

Plaza-Manzano et al. [21] carried out a systematic review and meta-analysis on the benefits of percutaneous nerve stimulation for the treatment of pain and related disability in musculoskeletal pain pathologies. The authors reported it may reduce the pain level but not the related disability in such disorders, although the level of scientific evidence on the studies was low. Regarding chronic LBP, there are a limited number of investigations studying the application of this technique in such pathology. In a previous study, San-Emeterio-Iglesias et al. [15] evaluated the best anatomical point of the sciatic nerve pathway to apply the US-guided PNM technique to achieve clinical benefits in LBP patients. They showed that all intervention point caused improvement of pain level, hip passive ROM, and balance in such patients. However, to the best of the authors’ knowledge, no research has studied the effects of US-guided PNM targeting the sciatic nerve to increase hip muscle strength in LBP patients, and treat one of the origins of this pathology, that is, hip muscle disbalance. Therefore, the main aim of this study was to investigate the effect of the US-guided PNM technique on hip muscle strength in people with LBP. In a like manner to a previous study [15], another purpose was to discover whether the location along the sciatic nerve where percutaneous neuromodulation was applied could influence the change of strength response in these patients.

## 2. Materials and Methods

### 2.1. Design

A prospective, randomized, controlled pilot study (NCT04590443) was carried out on LBP patients, according to the Consolidated Standards of Reporting Trials (CONSORT) guidelines.

### 2.2. Institutional Review Board

The local ethics committee approved the study, which complied with all the principles set forth in the Declaration of Helsinki. All participants signed informed written consent forms to participate in this study.

### 2.3. Participants

A convenience sample of 60 LBP sufferers diagnosed by a specialized doctor from a private clinic were recruited for this study. Participants were divided randomly (by a researcher) into three groups, each composed of 20 subjects, depending on the anatomical location for a specific stimulation: in group A, patients were stimulated in the proximal posterior thigh; in group B, patients were stimulated in the mid posterior thigh; and in group C, patients were stimulated in the distal posterior thigh. The reason for performing sciatic neuromodulation by localization was due to the fact that there are methodological questions regarding the development of this technique that are still not clear, but that are successfully applied during clinical practice. That is why the authors considered it important to demonstrate different manners that therapists apply in this technique, in order to establish adequate and scientifically justified treatment protocols.

**Figure 1:**
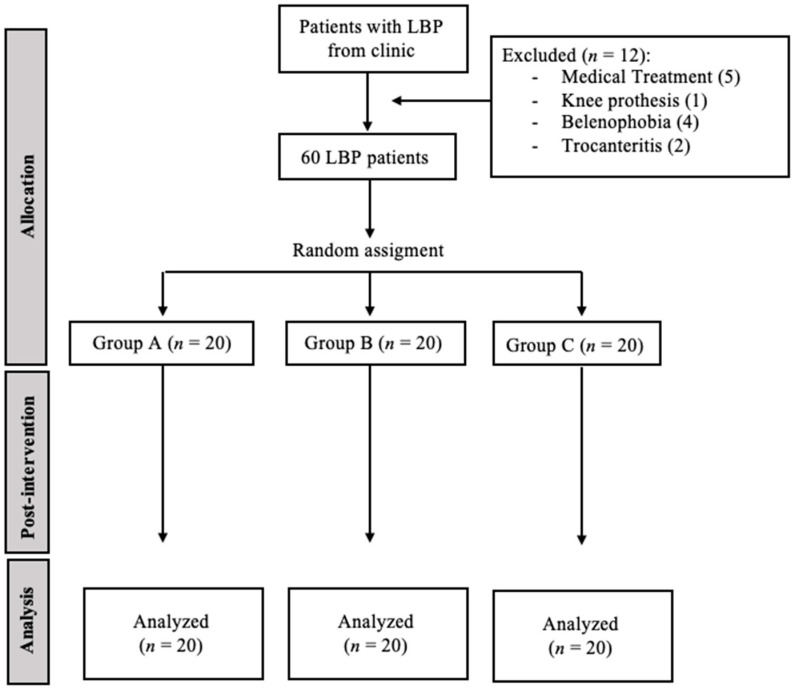
Provides a flowchart of subject recruitment during the study.

The recruitment dates were from 20 October 2020 to 10 July 2021. Figure 1 shows a flowchart of participant recruitment during the research.

According to the NIH Task Force on Research Standards for Chronic LBP (CLBP), patients who had referred nonspecific lumbar pain for at least the previous six months were recruited [22]. Specifically, patients must meet the following criteria in the clinical examination: nonspecific lumbar and/or gluteal pain; absence of sciatic neuropathy or radiculopathy on clinical symptoms; and a negative Lasegue test. In addition, subjects who were over 18 years of age [23], with pain intensity at least 3/10 on a numerical rating scale (NRS) [24], and one hip with limited passive internal rotation, as chronic LBP may be naturally linked to this hip biomechanical compensation [25], were also included. The exclusion criteria were: to be receiving any medical treatment; any musculoskeletal or neuropathic pathology [26]; previous lower extremity or back surgery; electrophysiological alteration attributable to another peripheral nerve; any contraindications to electrical therapy or needling [11]; pregnants; and epilepsy.

### 2.4. Outcome Measures

The following sociodemographic data were noted: age, weight, height, body mass index (BMI), sex, dominant side, and intervention side.

In line with the previous literature [13,15], which showed that the changes in the clinical variables caused by an isolated US-guided PNM intervention lasted one week, the researchers decided that all the variables should be recorded, before the intervention and one week after the intervention, by two qualified therapists blinded to the participant assignment group.

The clinical outcomes were one-week changes in pain intensity in the low back calculated with a numerical rating scale (0, no pain; 10, worst imaginable pain) and related disability measured with a specific LBP questionnaire. The Oswestry Disability Index (ODI) was used to assess lumbar back disability [27]. A 10-item scale (pain intensity, personal care, lifting, walking, sitting, standing, sleeping, work, social life, and traveling) with six levels composed the questionnaire. The final result is classified as minimal disability (0–20), moderate disability (21–40), severe disability (41–60), crippled (61–80), and bed-bound or exaggerating their symptoms (81–100).

The functional variables were one-week changes in hip IR-ROM and hip muscle strength.

- *Hip passive internal rotation range of motion* (*IR-ROM*). The authors only recorded the hip IR-ROM because it is the only one that is related to low back pain [6]. The hip passive IR-ROM was measured bilaterally. Each test was carried out three times and the average chosen as the final score. All measurements were performed by two testers, using a hand-held universal goniometer [28]. Tester 1 controlled the patient’s correct position and performed the passive hip movements and tester 2 measured and noted the hip ROM scores. To analyze hip IR, all individuals were positioned prone on a treatment table with the hip in neutral, the knee flexed to 90° and the pelvis stabilized with a belt, and the contralateral limb was placed in approximately 20° of abduction. The tester measured the ROM by bringing the limb to a firm end point of IR. Each test was carried out three times and the average calculated as the final score. Intra-rater reliability was established in the first 15 volunteers as sufficient for clinical measurement. The intraclass correlation coefficient (ICC) and the standard error of measurements (SEM) were 0.91 and 2.72, respectively, for the hip IR-ROM variable, indicating high reliability.
- *Hip muscle strength.* The hip muscle strength assessments of the hip abduction, external rotation, internal rotation, flexion and extension, were analyzed using a hand-held dynamometer (HHD) (Power Track II Commander/JTECH Medical, Salt Lake City, Ut, USA) [29]. Each measurement was taken bilaterally. The hip flexion and abduction strength were measured in the supine position, the hip extension strength was measured in the prone position, and the hip internal rotation-external rotation strength was measured in the prone position, and in the sitting position. The test positions were chosen based on clinical practice [30], and all strength tests were isometric strength tests, according to previous studies [31]. All patients were instructed in the procedures, and that they should achieve one isometric sub-maximal contraction into the tester’s hand. Each test was carried out three times and the average calculated as the final score. Intra-rater reliability was established in the first 15 volunteers as sufficient for clinical measurement. The ICC and SEM were 0.88 and 4.1, respectively, for the abduction variable, indicating good reliability; 0.91 and 3.5, respectively, for the external rotation variable, indicating high reliability; 0.93 and 4.0, respectively, for the internal rotation variable, indicating high reliability; 0.85 and 2.2, respectively, for the flexion variable, indicating good reliability; and 0.89 and 1.5, respectively, for the extension variable, indicating good reliability.

### 2.5. Ultrasound-Guided PNM Procedure

**Figure 2:**
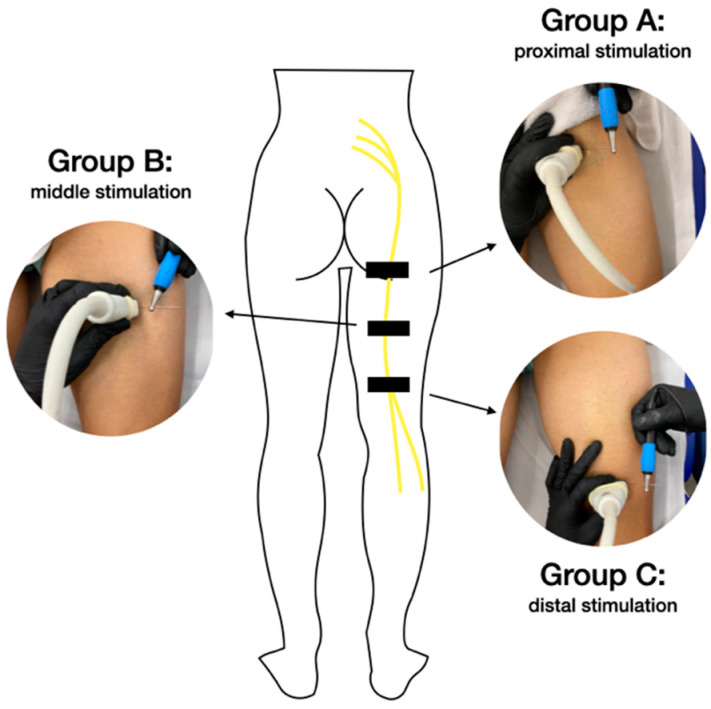
Ultrasound-guided percutaneous neuromodulation. Proximal stimulation of the sciatic nerve (Group A); sciatic nerve stimulation in the middle of the thigh (Group B); distal stimulation of the sciatic nerve (Group C).

An isolated percutaneous electrical stimulation intervention was applied to all patients, by a qualified physiotherapist with over 10 years of training in invasive techniques and ultrasound evaluations. The therapeutic procedure was carried out on the sciatic nerve because it originates in the lumbosacral plexus that innervates the lumbosacral area and lower limbs. To this aim, the skin surface was cleaned previously with chlorhexidine. Subsequently, the therapist inserted an acupuncture needle, using a long axis approach, until it reached the perineurium of the sciatic nerve (Figure 2). The sciatic nerve was located using a probe placed (in a transverse axis) in an ultrasound machine (Logiq E, GE Healthcare^®^, Waukesha, WI, USA) with a high-frequency linear US transducer (12 L). Following previous authors [15,19], the neuromodulation procedure was based on the application of a biphasic continuous waveform, at a low (3 Hz) frequency, pulse width 250 µs and at a sufficient intensity to see muscle contraction: 10 stimulations with a duration of 10 s, with a 10 s rest period between each stimulation. For the neuromodulation intervention, the equipment used was the Physio Invasiva^®^, (CEO120; PRIM Physio, Madrid, Spain), using the PES option, and the location of the needle was different for each group (Figure 2): for group A, the needle, measuring 0.30 mm × 75 mm, was placed in the proximal posterior thigh; for group B, the needle, measuring 0.30 mm × 60 mm, was placed in the mid posterior thigh; and for group C, the needle, measuring 0.30 mm × 40 mm, was placed in the distal posterior thigh. All the participants lay prone with their feet outside the therapeutic table.

The stimulation was carried out on the limb that had the restricted hip IR-ROM by the decision of the researchers, and according to the literature [6,15].

### 2.6. Data Analysis

The SPSS 23.0 software (SPSS Inc. IBM Chicago, IL, USA) was used for data analysis. The Kolmogorov-Smirnov test was run for the normality assumption. The one-way analysis of variance (ANOVA) was employed to assess the sociodemographic data among groups. Two-way ANOVA was used to calculate between group difference, with time (baseline, one week) as a within-subject factor and group (A, B, and C) as a between-subject factor. Moreover, the Mauchly test was developed to assess sphericity, and the Greenhouse-Geisser correction was employed when the sphericity assumption was not fulfilled. The level of significance was set at *p* < 0.05 with an α error of 0.05 (95% confidence interval) and a desired power of 80% (β error of 0.2).

The intraclass correlation coefficient (ICC) and the standard error of measurements (SEM) were used to determine the intra-researcher reliability of the measurements.

## 3. Results

Throughout the study, no adverse events were recorded. Table 1 shows that there were no significant baseline differences among the groups in any sociodemographic data. Table 2 shows that intra-group (time) values reported statistically significant differences (*p* = 0.001, Eta^2^ = 0.806 and *p* = 0.001, Eta^2^ = 0.727, respectively) and inter-group (treatment × time) differences were not found for the pain variable. However, there were significant differences for ODI (*p* = 0.021), but the effect size was small (Eta^2^ = 0.1) and the minimum important difference was not reached (points = 17) to be clinically meaningful [32]. The ODI values obtained by each group corresponded to the category “minimal disability”. The IR-ROM variable revealed statistically significant differences (*p* = 0.001). However, inter-group (treatment × time) interaction did not reveal significant differences for any variable. Regarding Table 3, intra-group (time) values reported statistically significant differences for all strength variables (*p* = 0.001). Inter-group (treatment x time) differences were only found for flexion strength in the non-intervention limb (*p* = 0.029), although the effect size was small (Eta^2^ = 0.1).

## 4. Discussion

The main finding was that the application of an isolated intervention of the US-guided PNM technique increased the hip muscle strength in patients with chronic LBP, in addition to other known effects such as pain and IR-ROM improvement. According to previous study [20], the hypothesis would be that the PNM would mainly stimulate the fast-twitch fibers and the inhibitory influences physiologically characteristic of the maximum voluntary efforts would be delayed during the application, providing a more intense contraction of the muscles that are being stimulated during muscular tests. This clinical improvement was maintained for at least one week, consistent with current evidence [13,15]. Therefore, researchers may affirm that neural stimulation, specifically US-guided PNM, is a minimally invasive intervention that has an interesting therapeutic potential for LBP sufferers. This means that hip disbalance (limited IR-ROM and muscle weakness), characteristic of LBP patients, may be treated by applying this technique by therapists due to the gain in muscle strength as well as other clinical variables that may be affected for these musculoskeletal pain disorders. Similar to previous study [15], the choice of a different anatomical point to stimulate the sciatic nerve did not influence the improvement of strength in these patients due to all groups improved hip muscle strength, but there was no difference between them. This clinical finding responds to a methodological question that therapists must take into account when applying the technique and thus, that there is a consensus.

A hallmark of chronic LBP is altered biomechanics of the lumbopelvic-hip zone [33], characterized by two aspects: on the one hand, gluteus medius weakness [7], and on the other hand, limited hip IR-ROM [6]. This mean that patients with LBP show a different hip muscle activation compared to healthy subjects (distal-to-proximal and proximal-to-distal muscle recruitment pattern, respectively) [34]. That is why physical exercise has become a main tool for the treatment of these patients. Recently, a systematic review [35] investigated the impact of an exercise program on pain and related functionality in patients with LBP, compared to other physiotherapeutic treatments. This review suggested that exercise therapy may be effective for the management of chronic LBP as well as placebo, usual care, no treatment, or other traditional treatments for pain and for functional limitations. However, if they considered to compare all groups, these differences were not clinically relevant [35].

The authors suggest that the search for other therapeutic tools complementary to therapeutic exercise may be an interesting line of future research for the treatment of LBP sufferers. Even though most prior research in US-guided PNM has focused on pain treatment in patients [4,5,6,7,8,9,10,11,12], others studies have explored the benefits of the US-guided PNM technique for other variables in healthy people. For example, De-la-Cruz-Torres et al. [18] demonstrated that applying this procedure on the sciatic nerve may increase hamstring flexibility; De-la-Cruz-Torres et al. [16,17] also reported an increase in flexor hallucis longus muscle performance in dancers; Álvarez-Prats et al. [19] showed an improvement in quadriceps muscle strength after performing the electrical stimulation on the femoral nerve; and Gallego-Sendarrubias et al. [20] suggested an increase in performance skills in soccer players, applying the neuromodulation procedure on the femoral nerve before specific strength training. Our results are consistent with these previous studies due to the improvement in pain, the IR-ROM, and muscle strength that we obtained when applying the US-guided PNM in LBP sufferers. In addition, our data are also in agreement with previous studies that analyzed the methodological question about the best point of electrical nerve stimulation to obtain therapeutic benefit [15,18]. The authors suggest that the success of US-guided PNM in LBP patients is assured, independent of the anatomical point of sciatic stimulation.

In this line, this is the first study applying the US-guided PNM technique with the aim of improving strength in the hip muscle in patients with LBP, while managing to improve the two biomechanical alterations characteristic of such patients: passive IR-ROM and muscle weakness. The authors reaffirm the therapeutic potential of the US-guided PNM technique, due to the extensive benefits for a multitude of important study variables in people’s daily lives. So, the relevance of this finding for clinical practice is that the US-guided PNM procedure may be prescribed as an important treatment modality in response to chronic LBP concerns. Based on our results, the US-guided PNM technique may be a complementary intervention to exercise therapy for improving hip muscle strength in LBP sufferers and therefore, relieving pain and improving related functional limitations.

The authors recognize that this study has several limitations: first, this neuromodulation technique was performed independently; usually, physiotherapists apply different methods in the same therapeutic appointment to treat patients referred with chronic LBP in clinical practice. Future studies that combine different procedures including the US-guided PNM technique are needed. Second, this study did not have a group control. Previous research demonstrated the non-application of any treatment [12], the application of the needle without current [18], or the use of other physiotherapy treatments [16]. Third, the authors only studied nonspecific low back pain. Further investigations should record information from patients’ pathology and evaluate its feasible influence on the outcomes. Fourth, the authors only measured the immediate effects (one week) because their objective was to analyze the benefit of the technique on the strength variable. Typically, neuromodulation techniques are applied to treat painful syndromes with the aim of relieving pain. However, the potential of this technique is greater and the authors wanted to highlight it. Research that analyzes the long-term effects are needed. Finally, the sample size was small; however, the authors consider that the findings of this study may provide an innovate perspective for the treatment of patients with chronic LBP, complementary to other medical treatments. The use of a newer technique also requires further research.

### Clinical Applications

The authors suggest that the main clinical application of this study is that US-guided PNM may be considered as a neuromodulation technique to treat chronic LBP. This procedure is able to produce improvements in multitude clinical variables, including pain.

## 5. Conclusions

The conclusion of this study was that the application of an isolated intervention of the US-guided PNM technique improved hip strength, the ROM, and the overall level of pain in LBP sufferers, independent of the anatomical point of sciatic stimulation.

## Figures and Tables

**Table 1:** Sociodemographic data of the sample.

Data	Total (*n* = 60)	Group A (*n* = 20)	Group B (*n* = 20)	Group C (*n* = 20)	*p*-Value
Age (years)	41.98 (12.9)	42.40 (12.1)	43.9 (15.3)	39.65 (11.3)	0.583
Weight (kilograms)	71.31 (12.2)	73.3 (13.2)	70.15 (13.1)	70.5 (10.4)	0.678
Height (meters)	1.71 (0.07)	1.71 (0.09)	1.69 (0.07)	1.71 (0.06)	0.728
BMI (kg/m^2^)	24.23 (3.26)	24.56 (3.23)	24.22 (3.84)	23.9 (2.76)	0.823
Gender (F/M)	30/30	10/10	10/10	10/10	N/A
Dominant side (R/L)	47/13	17/3	15/5	15/5	N/A
Intervention side (R/L)	26/34	9/11	7/13	10/10	N/A

Abbreviations: BMI, body mass index; F, female; L, left; M, male; R, right.

**Table 2:** Pain level, Oswestry Disability Index (ODI) and internal rotation range of motion for both limbs (IR-ROM) intrasubject treatment effects.

				Intrasubject Treatment Effects	
Measure	Group A	Group B	Group C	Time Value F (Df); *p* (Eta^2^)	Treatment × Time F (Df); *p* (Eta^2^)
**Pain (NRS)**				F (1, 57) = 237.12; *p* = 0.001 (0.81)	F (1, 57) = 1.29; *p* = 0.28 (0.05)
Baseline	4.85 ± 1.3	5.50 ± 0.9	5.30 ± 1.3		
1 week	1.90 ± 1.2	2.20 ± 1.3	2.75 ± 1.7		
**ODI (%)**				F (1, 57) = 151.47; *p* = 0.001(0.73)	F (1, 57) = 4.13; *p* = 0.02 (0.13)
Baseline	16.3 ± 5.2	19.3 ± 9.9	17.4 ± 7.7		
1 week	8.1 ± 4.8	6.7 ± 5.7	9.8 ± 7.4		
**IR prone** **Non-Intervention limb (°)**				F (1,57) = 12.45; *p* = 0.001(0.18)	F (1,57) = 1.18; *p* = 0.31 (0.04)
Baseline	36.7 ± 5.4	40.1 ± 7.3	40.9 ± 5.2		
1-week	38.1 ± 5.2	40.7 ± 6.2	41.5 ± 5.1		
**IR prone** **Intervention limb (°)**				F (1,57) = 252.20; *p* = 0.001(0.82)	F (1,57) = 1.18; *p* = 0.31 (0.04)
Baseline	29.6 ± 5.4	31.3 ± 6.5	33.4 ± 5.5		
1-week	38.3 ± 5.5	39.2 ± 5.4	40.2 ± 4.8		

Values are mean ± SD unless otherwise indicated. Abbreviations: IR, internal rotation, NRS, numerical rating scale; ODI, Oswestry Disability Index; F, F-test; Df, degrees of freedom.

**Table 3:** Flexion, extension, external and internal rotation, abduction strength for both limbs intrasubject effects.

				Intrasubject Effects	
Measure (Kgf)	Group A	Group B	Group C	Time Value F (Df); *p* (Eta^2^)	Treatment × Time F (Df); *p* (Eta^2^)
**Flexion Non-Intervention limb**				F (1,57) = 111.95; *p* = 0.001 (0.66)	F (1,57) = 3.77; *p* = 0.03 (0.12)
Baseline	15.2 ± 3.0	13.0 ± 3.7	14.0 ± 2.8		
1-week	17.7 ± 3.7	15.6 ± 3.2	15.4 ± 2.3		
**Flexion Intervention limb**				F (1,57) = 129.48; *p* = 0.001 (0.70)	F (1,57) = 0.25; *p* = 0.78 (0.01)
Baseline	14.3 ± 3.3	12.1 ± 3.8	12.8 ± 3.1		
1-week	17.8 ± 5.1	15.5 ± 3.3	15.8 ± 3.7		
**ER sitting Non-Intervention limb**				F (1,57) = 83.99; *p* = 0.001 (0.60)	F (1,57) = 0.50; *p* = 0.61 (0.02)
Baseline	11.8 ± 3.1	9.8 ± 2.7	10.9 ± 2.7		
1-week	13.8 ± 3.6	11.6 ± 2.5	12.4 ± 2.8		
**ER sitting Intervention limb**				F (1,57) = 101.48; *p* = 0.001 (0.64)	F (1,57) = 0.51; *p* = 0.601(0.018)
Baseline	10.8 ± 2.7	9.5 ± 2.7	10.1 ± 2.6		
1-week	13.7 ± 3.6	12.0 ± 2.8	12.4 ± 2.6		
**IR sitting Non-Intervention limb**				F (1,57) = 57.39; *p* = 0.001 (0.50)	F (1,57) = 0.57; *p* = 0.57 (0.02)
Baseline	12.3 ± 2.2	10.7 ± 2.5	11.0 ± 2.5		
1-week	14.9 ± 3.9	13.3 ± 3.3	12.9 ± 2.7		
**IR sitting Intervention limb**				F (1,57) = 71.49; *p* = 0.001 (0.56)	F (1,57) = 0.84; *p* = 0.43 (0.03)
Baseline	11.5 ± 2.1	9.4 ± 2.0	10.4 ± 2.7		
1-week	14.7 ± 3.1	13.0 ± 3.5	12.8 ± 3.4		
**ER prone Non-Intervention limb**				F (1,57) = 24.64; *p* = 0.001 (0.30)	F (1,57) = 0.53; *p* = 0.59 (0.02)
Baseline	12.2 ± 2.9	10.4 ± 2.2	11.5 ± 3.3		
1-week	13.4 ± 3.5	11.7 ± 2.1	12.3 ± 2.6		
**ER prone Intervention limb**				F (1,57) = 89.63; *p* = 0.001 (0.61)	F (1,57) = 0.13; *p* = 0.88 (0.004)
Baseline	11.5 ± 2.2	10.1 ± 2.6	10.3 ± 2.4		
1-week	13.6 ± 3.3	12.4 ± 2.5	12.4 ± 2.8		
**IR prone Non-Intervention limb**				F (1,57) = 53.84; *p* = 0.001 (0.49)	F (1,57) = 0.85; *p* = 0.43 (0.03)
Baseline	10.5 ± 2.4	9.7 ± 2.7	9.5 ± 2.2		
1-week	12.7 ± 3.2	11.1 ± 2.4	11.2 ± 2.3		
**IR prone Intervention limb**				F (1,57) = 166.47; *p* = 0.001 (0.75)	F (1,57) = 2.06; *p* = 0.14 (0.07)
Baseline	9.1 ± 2.3	8.5 ± 2.4	8.8 ± 2.3		
1-week	12.8 ± 3.7	11.5 ± 2.6	11.4 ± 2.4		
**Abduction Non-Intervention limb**				F (1,57) = 71.03; *p* = 0.001 (0.55)	F (1,57) = 1.36; *p* = 0.27 (0.04)
Baseline	12.5 ± 2.1	11.5 ± 3.0	12.1 ± 2.3		
1-week	14.8 ± 3.5	13.8 ± 2.6	13.6 ± 2.6		
**Abduction Intervention limb**				F (1,57) = 111.79; *p* = 0.001 (0.66)	F (1,57) = 1.07; *p* = 0.35 (0.04)
Baseline	11.3 ± 1.9	10.6 ± 2.9	10.7 ± 2.3		
1-week	15.3 ± 3.6	14.2 ± 2.9	13.6 ± 3.0		
**Extension Non-Intervention limb**				F (1,57) = 61.86; *p* = 0.001 (0.52)	F (1,57) = 2.29; *p* = 0.11 (0.07)
Baseline	19.4 ± 4.3	16.1 ± 3.5	17.5 ± 4.3		
1-week	24.9 ± 9.5	19.9 ± 4.0	20.3 ± 4.6		
**Extension Intervention limb**				F (1,57) = 109.80; *p* = 0.001 (0.66)	F (1,57) = 1.11; *p* = 0.34 (0.0)
Baseline	17.4 ± 4.6	15.0 ± 3.2	15.9 ± 4.2		
1-week	24.6 ± 9.5	20.9 ± 4.3	21.0 ± 4.5		

Values are mean ± SD unless otherwise indicated. Abbreviations: ER, external rotation; IR, internal rotation; F, F-test; Df, degrees of freedom.

## Data Availability

The data presented in this study are available on request from the corresponding author. The data are not publicly available due to ethical condition.

## References

[1] Bell J.A., Burnett A. (2009). Exercise for the primary, secondary and tertiary prevention of low back pain in the workplace: A systematic review. J. Occup. Rehabil..

[2] GBD 2016 Disease and Injury Incidence and Prevalence Collaborators (2017). Global, regional, and national incidence, prevalence, and years lived with disability for 328 diseases and injuries for 195 countries, 1990–2016: A systematic analysis for the Global Burden of Disease Study. Lancet.

[3] Borenstein D.G. (2001). Epidemiology, etiology, diagnostic evaluation, and treatment of low back pain. Curr. Opin. Rheumatol..

[4] Chou R., Qaseem A., Snow V. (2007). Diagnosis and treatment of low back pain: A joint clinical practice guideline from the American College of Physicians and the American Pain Society. Ann. Intern. Med..

[5] Kendall K.D., Schmidt C., Ferber R. (2010). The relationship between hip-abductor strength and the magnitude of pelvic drop in patients with low back pain. J. Sport Rehabil..

[6] Sadeghisani M., Manshadi F.D., Kalantari K.K., Rahimi A., Namnik N., Karimi M.T., Oskouei A.E. (2015). Correlation between Hip Rotation Range-of-Motion Impairment and Low Back Pain. A Literature Review. Ortop. Traumatol. Rehabil..

[7] Cooper N.A., Scavo K.M., Strickland K.J., Tipayamongkol N., Nicholson J.D., Bewyer D.C., Sluka K.A. (2016). Prevalence of gluteus medius weakness in people with chronic low back pain compared to healthy controls. Eur. Spine J..

[8] Reiman M.P., Bolgla L.A., Loudon J.K. (2012). A literature review of studies evaluating gluteus maximus and gluteus medius activation during rehabilitation exercises. Physiother. Theory Pract..

[9] Gilmore C.A., Kapural L., McGee M.J., Boggs J.W. (2019). Percutaneous Peripheral Nerve Stimulation (PNS) for the Treatment of Chronic Low Back Pain Provides Sustained Relief. Neuromodulation.

[10] Cohen S., Gilmore C., Kapural L., Hanling S., Plunkett A., McGee M., Boggs J. (2009). Percutaneous Peripheral Nerve Stimulation for Pain Reduction and Improvements in Functional Outcomes in Chronic Low Back Pain. Mil. Med..

[11] Valera-Garrido F., Minaya-Muñoz F. (2016). Fisioterapia Invasiva.

[12] De-la-Cruz-Torres B., Abuín-Porras V., Navarro-Flores E., Calvo-Lobo C., Romero-Morales C. (2021). Ultrasound-Guided Percutaneous Neuromodulation in Patients with Chronic Lateral Epicondylalgia: A Pilot Randomized Clinical Trial. Int. J. Environ. Res. Public Health.

[13] García-Bermejo P., De-la-Cruz-Torres B., Romer-Morales C. (2020). Ultrasound-guided percutaneous neuromodulation in patients with unilateral anterior knee pain: A randomised clinical trial. Appl. Sci..

[14] Fernández-de-Las-Peñas C., Arias-Buría J.L., El Bachiri Y.R., Plaza-Manzano G., Cleland J.A. (2020). Ultrasound-guided percutaneous electrical stimulation for a patient with cubital tunnel syndrome: A case report with a one-year follow-up. Physiother. Theory Pract..

[15] San-Emeterio-Iglesias R., Minaya-Muñoz F., Romero-Morales C., De-la-Cruz-Torres B. (2021). Correct Sciatic Nerve Management to Apply Ultrasound-Guided Percutaneous Neuromodulation in Patients with Chronic Low Back Pain: A Pilot Study. Neuromodulation.

[16] De-la-Cruz-Torres B., Barrera-García-Martín I., Albornoz-Cabello M. (2019). Immediate effects of ultrasound-guided percutaneous neuromodulation versus physical exercise on performance of the flexor hallucis longus muscle in professional dancers: A randomised clinical trial. Acupunct. Med..

[17] De-la-Cruz-Torres B., Barrera-García-Martín I., Romero-Morales C. (2020). Comparative Effects of One-Shot Electrical Stimulation on Performance of the Flexor Hallucis Longus Muscle in Professional Dancers: Percutaneous Versus Transcutaneous?. Neuromodulation.

[18] De-la-Cruz-Torres B., Carrasco-Iglesias C., Minaya-Muñoz F., Romero-Morales C. (2020). Crossover effects of ultrasound-guided percutaneous neuromodulation on contralateral hamstring flexibility. Acupunct. Med..

[19] Álvarez-Plats D., Carvajal-Fernández O., Pérez-Mellada N., Minaya-Muñoz F. (2019). Changes in Maximal Isometric Quadriceps Strength after the Application of Ultrasound-Guided Percutaneous Neuromodulation of the Femoral Nerve: A Case Series. Rev. Fisioter Invasiva.

[20] Gallego-Sendarrubias G.M., Arias-Buría J.L., Úbeda-D’Ocasar E., Hervás-Pérez J.P., Rubio-Palomino M.A., Fernández-de-Las-Peñas C., Valera-Calero J.A. (2021). Effects of Percutaneous Electrical Nerve Stimulation on Countermovement Jump and Squat Performance Speed in Male Soccer Players: A Pilot Randomized Clinical Trial. J. Clin. Med..

[21] Plaza-Manzano G., Gómez-Chiguano G.F., Cleland J.A., Arías-Buría J.L., Fernández-de-Las-Peñas C., Navarro-Santana M.J. (2020). Effectiveness of percutaneous electrical nerve stimulation for musculoskeletal pain: A systematic review and meta-analysis. Eur. J. Pain.

[22] Deyo R., Dworkin S.F., Amtmann D., Andersson G., Borenstein D., Carragee E., Carrino J., Chou R., Cook K., DeLitto A. (2014). Report of the NIH Task Force on research standards for chronic low back pain. Spine J..

[23] Carmona L., Ballina J., Gabriel R., Laffon A. (2001). The burden of musculoskeletal diseases in the general population of Spain: Results from a national survey. Ann. Rheum. Dis..

[24] Grande-Alonso M., Suso-Martí L., Cuenca-Martínez F., Pardo-Montero J., Gil-Martínez A., La Touche R. (2019). Physiotherapy Based on a Biobehavioral Approach with or without Orthopedic Manual Physical Therapy in the Treatment of Nonspecific Chronic Low Back Pain: A Randomized Controlled Trial. Pain Med..

[25] Avman M.A., Osmotherly P.G., Snodgrass S., Rivett D.A. (2019). Is there an association between hip range of motion and nonspecific low back pain? A systematic review. Musculoskelet. Sci. Pract..

[26] Grande-Alonso M., Muñoz-García D., Cuenca-Martínez F., Delgado-Sanz L., Prieto-Aldana M., La Touche R., Gil-Martínez A. (2020). Relationship between healthcare seeking and pain expansion in patients with nonspecific chronic low back pain. Peer J..

[27] Fairbank J.C., Pynsent P.B. (2000). The Oswestry disability index. Spine.

[28] Lea R.D., Gerhardt J.J. (1995). Range-of-motion measurements. J. Bone Joint Surg. Am..

[29] Thorborg K., Petersen J., Magnusson S.P., Hölmich P. (2010). Clinical assessment of hip strength using a hand-held dynamometer is reliable. Scand. J. Med. Sci. Sports.

[30] Pua Y.H., Wrigley T.W., Cowan S.M., Bennell K.L. (2008). Intrarater test–retest reliability of hip range of motion and hip muscle strength measurements in persons with hip osteoarthritis. Arch Phys. Med. Rehabil..

[31] Sisto S.A., Dyson-Hudson T. (2007). Dynamometry testing in spinal cord injury. J. Rehabil. Res. Dev..

[32] Maughan E.F., Lewis J.S. (2010). Outcome measures in chronic low back pain. Eur. Spine J..

[33] Nourbakhsh M., Arab A. (2002). Relationship between mechanical factors and incidence of low back pain. J. Orthop. Sports Phys. Ther..

[34] Nelson-Wong E., Poupore K., Ingvalson S., Dehmer K., Piatte A., Alexander S., Gallant P., McClenahan B., Davis A.M. (2013). Neuromuscular strategies for lumbopelvic control during frontal and sagittal plane movement challenges differ between people with and without low back pain. J. Electr. Kinesiol..

[35] Hayden J.A., Ellis J., Ogilvie R., Malmivaara A., van Tulder M.W. (2021). Exercise therapy for chronic low back pain. Cochrane Database Syst. Rev..

